# Pancréatite aiguë révélant un kyste hydatique du pancreas

**DOI:** 10.11604/pamj.2015.20.429.6734

**Published:** 2015-04-29

**Authors:** Mohamed Mattous, Soufiane Belabbes

**Affiliations:** 1 Service des Urgences du 4^ème^ Hôpital Militaire de Dakhla, Dakhla, Maroc; 2 Service d'Imagerie Médicale du 4^ème^ Hôpital Militaire de Dakhla, Dakhla, Maroc

**Keywords:** Kyste Hydatique, pancréatite, scanner, hydatid cyst, pancreatitis, CT scan

## Image en medicine

L'hydatidose est une maladie parasitaire très répandue dans le monde, notamment dans les pays d’élevage traditionnel. Sa localisation au niveau du pancréas est très rare, souvent révélée par un tableau de pancréatite aigue. Le diagnostic repose sur un faisceau d'arguments cliniques, biologiques et surtout radiologiques. Nous rapportons le cas d'une patiente âgée de 30 ans, sans antécédents pathologiques particuliers, admise aux urgences dans un tableau de douleurs épigastriques en barre avec vomissements, sans trouble de transit ni fièvre. Le bilan biologique a révélé une lipasémie à 10 fois la normale, et une hyperleucocytose à 13.500 éléments /mm^3^, permettant de poser le diagnostic d'une pancréatite aigue. Un scanner abdominal a été réalisé 48 heures après le début de la symptomatologie pour établir une stadification selon la classification de Balthazar, et a révélé une masse kystique de la tète du pancréas, bien limitée, arrondie, à paroi relativement fine et régulière, non rehaussée après injection du produit de contraste iodé. Cette masse renfermait des membranes flottantes faisant évoquer un kyste hydatique du pancréas. La sérologie hydatique réalisée était positive confirmant le diagnostic d'une pancréatite aigue compliquant un kyste hydatique du pancréas. La patiente fut adressée au service de chirurgie et a bénéficié d'une résection du dôme saillant associée à un drainage externe. Les suites post opératoires étaient simples.

**Figure 1:**
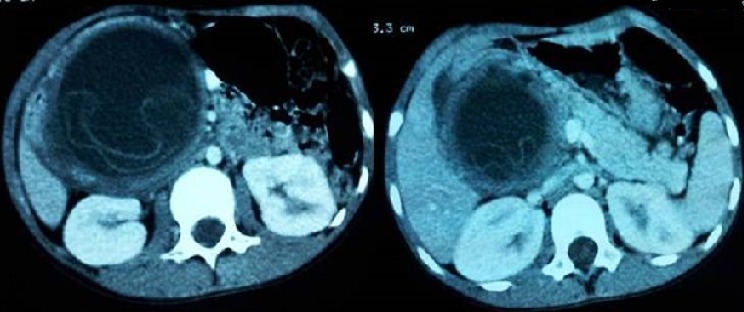
TDM abdominale avec injection de produit de contraste iodé, coupe axiale qui montre une masse kystique de la région pancréatique

